# A DICOM dataset for evaluation of medical image de-identification

**DOI:** 10.1038/s41597-021-00967-y

**Published:** 2021-07-16

**Authors:** Michael Rutherford, Seong K. Mun, Betty Levine, William Bennett, Kirk Smith, Phil Farmer, Quasar Jarosz, Ulrike Wagner, John Freyman, Geri Blake, Lawrence Tarbox, Keyvan Farahani, Fred Prior

**Affiliations:** 1 Department of Biomedical Informatics, University of Arkansas for Medical Sciences, Little Rock, Arkansas USA; 2 Arlington Innovation Center: Health Research, Virginia Tech, Arlington, Virginia USA; 3 Frederick National Laboratory for Cancer Research, Frederick, Maryland USA; 4 Center for Biomedical Informatics and Information Technology, National Cancer Institute, Bethesda, Maryland USA; 5 Department of Radiology, University of Arkansas for Medical Sciences, Little Rock, Arkansas USA

**Keywords:** Data publication and archiving, Image processing

## Abstract

We developed a DICOM dataset that can be used to evaluate the performance of de-identification algorithms. DICOM objects (a total of 1,693 CT, MRI, PET, and digital X-ray images) were selected from datasets published in the Cancer Imaging Archive (TCIA). Synthetic Protected Health Information (PHI) was generated and inserted into selected DICOM Attributes to mimic typical clinical imaging exams. The DICOM Standard and TCIA curation audit logs guided the insertion of synthetic PHI into standard and non-standard DICOM data elements. A TCIA curation team tested the utility of the evaluation dataset. With this publication, the evaluation dataset (containing synthetic PHI) and de-identified evaluation dataset (the result of TCIA curation) are released on TCIA in advance of a competition, sponsored by the National Cancer Institute (NCI), for algorithmic de-identification of medical image datasets. The competition will use a much larger evaluation dataset constructed in the same manner. This paper describes the creation of the evaluation datasets and guidelines for their use.

## Abstract (summary)

**Table:** 

Measurement(s)	Deidentification • Clinical Data
Technology Type(s)	data synthesis • digital curation
Factor Type(s)	imaging type
Sample Characteristic - Organism	Homo sapiens

Machine-accessible metadata file describing the reported data: 10.6084/m9.figshare.14802774

## Background & Summary

Open access or shared research data must comply with the Health Insurance Portability and Accountability Act (HIPAA) regulations that govern patient privacy. These regulations require the de-identification or removal of protected health information (PHI) and other personally identifiable information (PII) from datasets before they can be made publicly available. The Cancer Imaging Archive (TCIA)^1^ of the National Cancer Institute (NCI), is one of the largest and most trusted public archives of de-identified cancer images. Over the years, TCIA has developed image de-identification tools and protocols that combine automated and manual de-identification processes. This approach has proven effective for the de-identification of DICOM radiology imaging and digital pathology whole-slide imaging (WSI) submitted to TCIA.

The process of image de-identification and curation is time consuming, requires significant resources, and is prone to human fatigue and error. Automated image de-identification algorithms require evaluation before they can be deployed to process data for open access. This evaluation requires a robust dataset that can be used as a part of assessing image de-identification algorithms. We set out to develop a de-identification evaluation dataset to address that need. Because TCIA is one of the most mature imaging archives with an established and effective image de-identification method, we adopted the TCIA curation process as the current best practice in de-identification. Using TCIA and a newly developed toolset, we created an evaluation dataset by inserting synthetic PHI into already de-identified data.

While it is common to assume de-identification and anonymization are synonymous, in this document we follow Kushida *et al*.^2,3^ who make a clear distinction between these concepts: “De-identification of medical record data refers to the removal or replacement of personal identifiers so that it would be difficult to re-establish a link between the individual and his or her data. Anonymization refers to the irreversible removal of the link between the individual and his or her medical record data to the degree that it would be virtually impossible to reestablish the link.” Throughout this document, we will only deal with de-identification.

The evaluation dataset described in this data descriptor is a subset of a larger evaluation dataset created under contract for the National Cancer Institute. We published this subset on TCIA and explained it here to allow researchers to test their de-identification algorithms and promote standardized procedures for validating automated de-identification.

## Methods

**Fig. 1:**
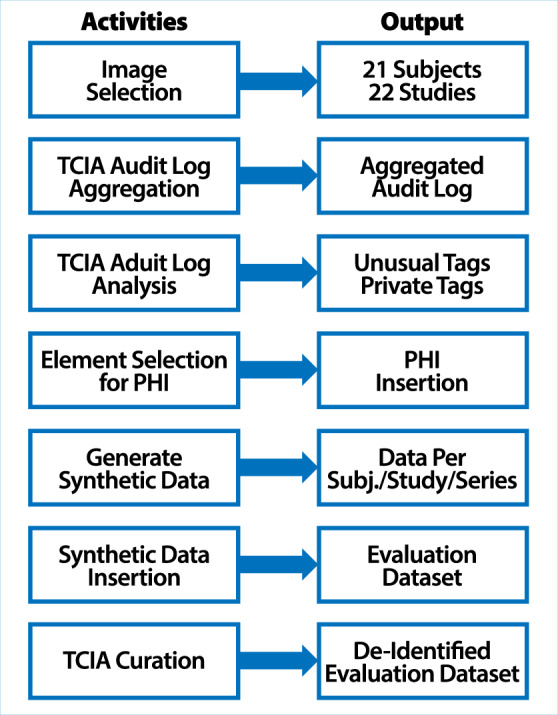
Schematic description of the processing steps involved in the creation of the evaluation dataset and de-identified evaluation dataset.

The full process of generating the evaluation dataset and de-identified evaluation dataset, which serves as an example result of applying a complete de-identification process to the evaluation dataset, is summarized in Fig. 1. Note that in this document, the terms “subject” and “patient” are used as synonyms.

### Images selected from TCIA

To build the evaluation dataset, we selected imaging studies from TCIA to represent a broad cross-section of the current TCIA public collections. Table 1 breaks down the content of the evaluation set into the total number of patients studies, series and images per modality, anatomy imaged by modality and manufacturers of imaging equipment used to collect the data. No images of heads were included to avoid subjects being identified by facial features^4,5^. The total image count for the evaluation set is 1,693 images that consist of 21 patients, 22 studies, and 26 series for a total of 609 MB of data.

**Table 1:** Evaluation Dataset Characterization.

DATA SET DESCRIPTION						
Modality	Patients	Studies	Series	Images	Anatomy (# Studies)	Manufacturer (# Studies)
CT	5	5	5	268	BLADDER (4) CHEST (1)	GE MEDICAL SYSTEMS (2) PHILIPS (1) SIEMENS (1) TOSHIBA (1)
MR	3	3	5	150	KIDNEY (2) PELVIS (1)	GE MEDICAL SYSTEMS (1) SIEMENS (2)
PT	5	5	6	1,203	[BLANK] (1) BREAST (2) EXTREMITY (2)	GE MEDICAL SYSTEMS (4) SIEMENS (1)
DX	4	4	4	10	CHEST (4)	GE MEDICAL SYSTEMS (1) PHILIPS (3)
CR	3	3	4	4	CHEST (2) UTERUS (1)	FUJIFILM (3)
MG	2	2	2	58	BREAST (2)	LORAD (1) VICTRE (1)
Total	21	22	26	1,693	22	22

This table describes the size of the dataset with totals for patients, studies, series, images, body part examined and manufacturers. (Note: VICTRE is not an equipment manufacturer, but a collection of synthetic image data). Imaging modalities are indicated using the DICOM conventions (CT = Computed Tomography, MR = Magnetic Resonance imaging, PT = Positron Emission Tomography, DX = Digital X-ray, CR = Computed Radiography, MG = Mammography).

### Implants

A handful of images containing medical implants were visually inspected for PHI by a trained member of TCIA’s curation team. It is important to visually inspect implant devices because they could contain a serial number that could be used to identify the patient^6^. If PHI is found, it should be removed or obscured in the image, and if not possible, then the image should not be published. In our selected images, we did not see any information that would warrant alteration or removal of images. Users of this dataset could be instructed to obscure the model numbers as a test of this capability, but normally they would not be required to make such modifications as model numbers do not constitute PHI since model numbers in general are not traceable back to an individual.

### DICOM Standard and Manufacturer’s Private Attributes Using Audit Logs

TCIA audit logs are updated whenever curators make any adjustments to DICOM information objects (including image headers) to remove potential PHI. These audit logs represent the complete provenance of the changes made to transform the submitted data into the published information objects^7^. The logs contain the before/after/replaced values of all DICOM standard Attributes and manufacturer’s Private Attributes^8^.

When DICOM data are submitted to TCIA, Private Attributes are de-identified according to the DICOM Retain Safe Private Option^9^ that allows for the retention of data stored in Private Attributes that do not hold PHI. Retention decisions are based on the extensive Private Attribute dictionary maintained by TCIA, which contains all the Private Attributes ever submitted to TCIA^8^. The dictionary also contains the process operation description (POD) used to modify the data in the Private Attribute to accomplish de-identification. The PODs are: (1) kept, (2) hashed, (3) off-set, (4) deleted, or (5) emptied. The choice of which POD to employ in a given instance is based on the Attribute Type and definition, e.g., DICOM unique identifiers (UIDs) are hashed, dates are off-set.

We stratified the coded data from the audit log by a combination of variables, including whether or not the DICOM Attribute is standard or private, DICOM Attribute description, and the TCIA process operation. A Pareto analysis^10^ was performed to determine the vital few data element/operation combinations that occur with the greatest frequency. Subsets of the results of this analysis can be found in Tables 2 and 3.

Table 2 lists examples of standard DICOM Attributes. Table 3 shows examples of Private Attributes; both tables list the Data Element tags (group and element number combination from the DICOM data dictionary) and the frequency counts of each. It should be noted that data fields listed do not always signify that PHI was seen during the de-identification process. Only that the potential for PHI existed and actions were taken to ensure that no PHI made it through the curation process.

**Table 2:** Unusual DICOM attributes containing PHI.

DICOM Tag	DICOM Description	Freq
<(0008,0041)>	Data Set Subtype	1
<(0018,1250)>	Receive Coil Name	2
<(0018,7006)>	Detector Description	3
<(0010,0021)>	Issuer of Patient ID	4
<(0032,1030)>	Reason for Study	5
<(0008,1080)>	Admitting Diagnoses Description	6
<(0032,1000)>	Scheduled Study Start Date	11
<(0018,0010)>	Contrast/Bolus Agent	15
<(0018,1401)>	Acquisition Device Processing Code	29
<(0018,1000)>	Device Serial Number	31
<(0008,1010)>	Station Name	33
<(0032,1060)>	Requested Procedure Description	37
<(0008,2111)>	Derivation Description	44
<(3006,0006)>	Structure Set Description	50
<(3006,0008)>	Structure Set Date	57
<(0032,4000)>	Study Comments	70
<(0010,21b0)>	Additional Patient History	76
<(0032,1070)>	Requested Contrast Agent	101
<(0008,1030)>	Study Description	297
<(0010,4000)>	Patient Comments	1192

The table displays examples of unusual DICOM attributes, and their frequency counts identified in the analysis of the TCIA audit logs.

**Table 3:** Private DICOM Attributes containing PHI.

DICOM Tag	DICOM Description	Freq
<(0027,“GEMS_IMAG_01”,33)>	ImagingOptions	1
<(3f01,“INTELERAD MEDICAL SYSTEMS”,03)>	SourceAE	1
<(7005,“TOSHIBA_MEC_CT3”,1c)>	Contrast/Bolus Agent for Series Record	1
<(0009,“GEMS_PETD_01”,37)>	Batch Description	2
<(0045,“GEMS_SENO_02”,26)>	MAOBuffer	2
<(0009,“FDMS 1.0”,92)>	KanjiDepartmentName	3
<(0009,“GEMS_IDEN_01”,30)>	ServiceId	4
<(0043,“GEMS_PARM_01”,80)>	Coil ID Data	8
<(0021,“SIEMENS MR SDS 01”,19)>	MR Phoenix Protocol	15
<(0023,“GEMS_STDY_01”,70)>	StartTimeSecsInFirstAxial	156

The table displays examples of Private DICOM Attributes, and their frequency counts identified in the analysis of the TCIA audit logs.

### Generation of synthetic data

Synthetic PHI data elements were generated using the Python package Faker (https://pypi.org/project/Faker, version 4.1.2). In addition to data elements one might expect to contain PHI, e.g., Patient Name and Address, we identified common Attributes, such as Study Description, which could potentially contain useful information while also containing PHI. These Attributes were selected for potential synthetic PHI insertion to demonstrate that deleting or emptying Attributes indiscriminately is not always the best solution, rather the information in the Attribute needs to be modified to retain important information while removing PHI.

### Selecting research critical fields and adherence to DICOM standard

In the DICOM standard, each Attribute is assigned a Type that specifies whether the Attribute is mandatory, optional, or conditional. The Attribute Type may be dependent on the modality of the image. The five Attribute Types are shown in Table 4.

We focused only on attributes that were Type 1 (attribute required, valid value required) and Type 2 (attribute required, value may be null). Type 1 C and 2 C attributes are conditional and require a determination if the conditions have been met that dictate whether the Data Element is a type 1 or 2. Therefore, no Type 1 C or 2 C attributes were modified with synthetic-PHI, although we retained Type 1 C and 2 C attributes in the image headers under the assumption that they were properly de-identified during initial TCIA curation. Also note, Attribute Types vary depending on the Service Object Pair (SOP) Class (modality), so we took this into account when generating our list of required Attributes.

**Table 4:** Attribute Types.

Type	Description
Type 1:	Required to be in the SOP Instance and shall have a valid value.
Type 2:	Required to be in the SOP Instance but may contain the value of “unknown”, or a zero length value.
Type 3:	Optional. May or may not be included and could be zero length.
Type 1C:	Conditional. If a condition is met, then it is a Type 1 (required, cannot be zero). If condition is not met, then the tag is not sent.
Type 2C:	Conditional. If condition is met, then it is a Type 2 (required, zero length OK). If condition is not met, then the tag is not sent.

The table displays Attribute Types as defined in the DICOM standard.

Table 5 shows a subset of the full list of Research Critical Fields we generated, showing the requirements for various DICOM Attributes for different modalities and the types and descriptions of each. The modality column signifies how the Attributes are treated based on modality. For fields where this entry is “All”, the type applies to all modalities. The tag column provides the DICOM group and element tag for the data element that encodes the Attribute, the Attribute column contains the name of the Attribute, the desc column provides a description and conditional requirements, and the Type column identifies the Attribute Type (1, 1 C, 2, or 2 C) as shown in Table 4.

**Table 5:** General and modality specific data Attributes and Types as specified in the DICOM standard.

Tag	Attribute	Type	Modality	Description
<(0008,0016)>	SOP Class UID	1	All	Uniquely identifies the SOP Class.
<(0008,0020)>	Study Date	2	All	Date the Study started.
<(0008,0060)>	Modality	1	All	Type of equipment that originally acquired the data used to create the images in this Series.
<(0010,0010)>	Patient’s Name	2	All	Patient’s full name.
<(0020,0060)>	Laterality	2C	All	Laterality of <(paired)> body part examined. Required if the body part examined is a paired structure and Image Laterality <(0020,0062)> is not sent.
<(0028,0004)>	Photometric Interpretation	1	CR	Specifies the intended interpretation of the pixel data.
<(0008,0008)>	Image Type	1	CT	Image identification characteristics.
<(0018,0060)>	KVP	2	CT	Peak kilo voltage output of the x-ray generator used
<(0008,0068)>	Presentation Intent Type	1	DX	Identifies the intent of the images that are contained within this Series.
<(0008,0070)>	Manufacturer	2	DX	Manufacturer of the equipment that produced the Composite Instances.
<(0028,0120)>	Pixel Padding Value	1C	DX	Required if Pixel Padding Range Limit (0028,0121) is present and either Pixel Data (7FE0,0010) or Pixel Data Provider URL (0028,7FE0) is present. May be present otherwise only if Pixel Data (7FE0,0010) or Pixel Data Provider URL (0028,7FE0) is present.
<(6000,3000)>	Overlay Data	1	DX	Overlay pixel data.
<(0018,1508)>	Positioner Type	1	MG	MAMMOGRAPHIC or NONE
<(0040,0318)>	Organ Exposed	1	MG	Organ to which Organ Dose (0040,0316) applies. BREAST
<(0028,0100)>	Bits Allocated	1	MR	Number of bits allocated for each pixel sample. Each sample shall have the same number of bits allocated.
<(0028,0101)>	Bits Stored	1	MR	Number of bits stored for each pixel sample. Each sample shall have the same number of bits stored.
<(0020,0032)>	Image Position <(Patient)>	1	PT	The x, y, and z coordinates of the upper left hand corner <(center of the first voxel transmitted)> of the image, in mm.
<(0020,0037)>	Image Orientation <(Patient)>	1	PT	The direction cosines of the first row and the first column with respect to the patient.
<(0008,0064)>	Conversion Type	1	SC	Describes the kind of image conversion

“All” applies to all modalities. Per the DICOM standard, Type 1 is required, Type 1 C is required if certain specified conditions are met, Type 2 is required but the value may be unknown (0 length), Type 2 C is a Type 2 conditional. DICOM Type 3 data elements are optional.

### Adoption of TCIA Curation as the best practice

**Fig. 2:**
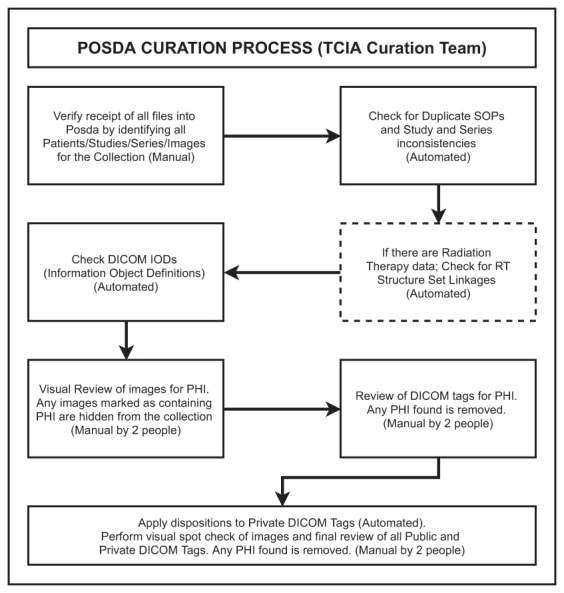
Schematic description of the standard TCIA Curation Workflow based on the Posda tool suite.

There is no clear definition of “important attributes” for secondary research in the research community. Many publications mention important DICOM attributes, but they were related more to the authors’ own research programs than a community-based consensus. Since TCIA is one of the most mature DICOM imaging archives, we adopted the TCIA curation process^7^, as illustrated in Fig. 2, and resultant dataset as the best practice on this issue.

### Creation of the evaluation dataset

To create the evaluation dataset, we deployed a process to re-identify DICOM images. For each image that was downloaded from TCIA for a specific patient (by Patient ID / Series ID / Study ID), we overwrote selected DICOM Attribute values with synthetic data. This repopulation of Attribute values was accomplished using version 0.7.5 of Posda^7^ (https://code.imphub.org/projects/PT/repos/oneposda), the open source package used for curation by TCIA. We created a file specifying the scope (Collection, Patient, Study, Series, Instance) as well as the operations to be performed, which are listed in Table 6. This file was then used by Posda to bulk edit the selected Attributes. For burn-in annotations (text within the pixel data), we extended these editing parameters to include both the text to be inserted and the coordinates of the location of the PHI on the image. Posda used the open source software package ImageMagick (https://imagemagick.org/index.php, version 7.0.9-7) to insert multiple lines of text into the Pixel Data.

**Table 6:** Re-Identification Operations.

Operation	Description
set_tag	Set specified tag to given value
delete_tag	Delete specified tag
shift_date	Shift date based on given value
substitute	Modifies tag with existing value
string_replace	Substitutes text within a tag
annotate_img	Burns given text at given coordinates

The table identifies operations utilized in the Posda tools to re-identify DICOM datasets with synthetic data.

### De-identified evaluation dataset

To create an example of how the evaluation dataset would look once re-de-identified using tools and procedures equivalent to those in current use by TCIA, a TCIA curation team that had no knowledge of the evaluation dataset creation process was tasked with the creation of a de-identified version of the evaluation dataset. This de-Identified evaluation dataset follows the standards outlined above as the best practice for de-identification.

### MIDI project dataset

The Medical Imaging De-Identification Initiative (MIDI), sponsored by the National Cancer Institute, produced a significantly larger evaluation dataset. After the creation of the full set, 21 records were split off to create the publishable evaluation dataset which is made available on TCIA and described in this publication. Please also note that we are unable to release some elements of the MIDI project due to the need to protect the integrity of the full dataset, which remains the property of the National Cancer Institute.

## Data Records

### MIDI-Evaluation collection

The evaluation dataset (containing synthetic PHI) and TCIA de-identified evaluation dataset (curated by TCIA) along with crosswalks for both patient IDs and DICOM UIDs between the two datasets have been published^11^. They may be accessed via the referenced DOI or via the TCIA collection browser as collection Pseudo-PHI-DICOM-Data (https://www.cancerimagingarchive.net/collections/).

## Technical Validation

To validate resultant curated datasets, an answer key was created to compare tag states between pre and post-curated datasets. An example of the answer key can be seen in Table 7. The answer key is driven by the actions listed in Table 8 along with action text (list of text retained or removed, etc.) for the various comparisons needed for evaluation. We wrote a Python evaluation script for comparing an answer key to a de-identified dataset. The inputs to the evaluation script are the answer key files along with a Patient ID Crosswalk containing a cross-reference between the old Patient ID and the new Patient ID and a UID Crosswalk for old to new UIDs, which are used for comparison per SOP class included in the collection.

When the TCIA curation team completed their curation task of generating the de-identified evaluation dataset, we compared that dataset to the answer key, and only expected discrepancies (e.g., new UID and Patient ID mapping) were found.

**Table 7:** Answer key format.

Scope	Tag	Tag Name	Action	Action Text
<Study>	<(0008,0050)>	<Accession Number>	<text_removed>	<[“20130912E245583”]>
<Study>	<(0008,0080)>	<Institution Name>	<text_removed>	<[“Treetop Medical Center”]>
<Study>	<(0008,0090)>	<Referring Physician’s Name>	<text_removed>	<[“ROBERTSON^JESSE”]>
<Study>	<(0008,1050)>	<Performing Physician’s Name>	<text_removed>	<[“PHILLIPS^JOHN”]>
<Study>	<(0008,0050)>	<Accession Number>	<text_removed>	<[“20130912E801911”]>
<Study>	<(0008,1030)>	<Study Description>	<text_removed>	<[“Billy Rogers”]>
<Study>	<(0008,1030)>	<Study Description>	<text_retained>	<[“XR CHEST AP PORTABLE”]>
<Study>	<(0008,0090)>	<Referring Physician’s Name>	<text_removed>	<[“BAILEY^THERESA”]>
<Study>	<(0008,1050)>	<Performing Physician’s Name>	<text_removed>	<[“SMITH^MARY”]>
<Patient>	<(0010,0020)>	<Patient ID>	<text_removed>	<[“6774825273”]>
<Patient>	<(0010,0010)>	<Patient’s Name>	<text_removed>	<[“ROGERS^BILLY”]>
<Patient>	<(0010,0030)>	<Patient’s Birth Date>	<text_removed>	<[“19430722”]>

This table shows the format of the answer key used to compare the results of de-identification to the original evaluation dataset. The answer key is based on TCIA de-identification standards and TCIA best practice.

**Table 8:** Answer Key actions.

Action	Description
tag_retained	The tag itself is retained and present in the DICOM dataset
text_notnull	The value of the tag is not null or zero length value
text_retained	The text specified was retained in the tag value
text_removed	The test specified was removed from the tag value
date_shifted	The date was shifted using the specified shift value
uid_changed	The UID was updated according to curation crosswalk
pixels_hidden	The pixels within coordinates specified are hidden

This table lists the actions used in the answer key to do the comparisons. Various actions were used such as tag retained to ensure a tag is not removed and date shifted to check whether a date was shifted using a particular shift value.

## Data Availability

Synthetic Protected Health Information (PHI) was generated using the Faker software package (https://pypi.org/project/Faker) and inserted into selected DICOM Attributes using an extended version of the Posda^7^ tool suite (https://code.imphub.org/projects/PT/repos/oneposda), the open source package used for curation and de-identification by TCIA. Posda incorporated the open source software package ImageMagick (https://imagemagick.org/index.php) to insert multiple lines of text into Pixel Data.
